# From Leksell’s Arc to El Salvador: The Thalamotomy of Pain and the Global Democratization of Functional Radiosurgery

**DOI:** 10.7759/cureus.97433

**Published:** 2025-11-21

**Authors:** Eduardo E Lovo

**Affiliations:** 1 Neurosurgery, Gamma Knife Program, International Cancer Center, Diagnostic Hospital, San Salvador, SLV

**Keywords:** artificial intelligence, chronic pain, democratization of science, el salvador, functional radiosurgery, gamma knife, lars leksell, thalamotomy

## Abstract

Lars Leksell’s pioneering vision of bloodless neurosurgery through radiosurgical precision revolutionized functional neurosurgery. This editorial revisits the historical and philosophical roots of radiosurgical thalamotomy for pain and explores its modern revival in low- and middle-income countries (LMICs), specifically in El Salvador. The integration of artificial intelligence (AI), connectomic targeting, and non-ablative dosing has redefined the possibilities of functional radiosurgery beyond traditional centers of innovation. At the Centro Internacional de Cáncer, we have implemented a wide range of radiosurgical targeting strategies, including sub-necrotic 90 Gy treatments to the thalamus and hypophysis for pain control. This experience highlights both the versatility of precision radiosurgery and its feasibility within resource-constrained environments. This article argues that the next frontier of functional neurosurgery lies not only in technical innovation but also in the ethical and global democratization of access. Were Leksell alive today, he would find his prophecy fulfilled in the convergence of data-driven precision and humanitarian purpose.

## Editorial

In 1951, Lars Leksell published “The stereotaxic method and radiosurgery of the brain,” introducing a concept that reshaped neurosurgery [1]. In those early experiments, focused X-ray beams converged on intracranial targets without craniotomy, envisioning bloodless operating rooms (ORs), reduced suffering, and broader treatment possibilities for intracranial disease. The method combined elegant physics with surgical vision: healing with beams instead of blades. That vision remains difficult for a small minority to embrace, at times even within our own specialty. Some colleagues, shaped by years of open surgical training, continue to favor craniotomy even when and where radiosurgery is equally available and offers a comparable therapeutic effect with a more favorable safety profile. Yet, this reflects familiarity more than opposition. Nevertheless, like steady and unstoppable waves of change, the broader trajectory of neurosurgery continues toward interventions that are increasingly precise and, when possible, bloodless, guided not by exposure and retraction, but by profound advancements in imaging that allow us to understand the functional interconnections of the brain, together with minimally invasive or entirely noninvasive applications of focused energy.

Among the first clinical applications of radiosurgery were thalamotomies for chronic pain [2]. Although the initial case series was small, it demonstrated the feasibility of creating a precisely placed lesion within the medial thalamus, with profound implications. Leksell’s stereotactic frame and center-of-arc geometry enabled sub-millimetric targeting of deep brain structures, less invasive than the standard surgery of the time. In 1967, these principles culminated in the Leksell Gamma Knife, a noninvasive instrument capable of producing focused ablative or non-ablative, modulatory lesions, an idea that would take decades to mature [3].

Pain as the genesis of functional radiosurgery

Pain has long served as both mystery and motivator in functional neurosurgery. Leksell’s early patients, including those with intractable cancer pain or trigeminal neuralgia, received radiosurgical lesions to the thalamus and mesencephalon [4]. These results suggested that carefully targeted irradiation could alter nociceptive transmission and affective processing, even when targets and lesions were not directly visible at the time; the technique was supported by strong anatomical knowledge.

Over subsequent decades, investigators such as Steiner, Young, Friehs, and Franzini refined target localization, dose, and patient selection [5-7]. Despite intermittent successes, radiosurgical approaches to pain remain under-investigated, often overshadowed by movement disorder indications and limited by access to advanced technology in low- and middle-income countries (LMICs).

From Stockholm to San Salvador

Seven decades later, the field has come full circle. In an era of growing attention to global health equity, Central America has begun to emerge as a site for studying and deploying radiosurgery for pain. The influence of Leksell’s work has reached widely, including our region.

At the International Cancer Center in San Salvador, our team sought to extend Leksell’s vision through a series of clinical and technical efforts from 2019 to 2025 [8-12]. These efforts included, to our knowledge, the first randomized controlled trial (RCT) of radiosurgery for mixed oncological pain using three targets (bilateral thalamus and hypophysis) with potentially subnecrotic doses of 90 Gy (Randomized controlled trial: Lovo et al. Randomized Controlled Trial of Triple Target Radiosurgery for Mixed Oncological Pain). Even a 50% effectiveness rate in patients with refractory pain would represent a meaningful advance for a noninvasive technique deliverable within hours, contingent on coordinated collaboration among clinicians, administrators, hospitals, society, government, and medical insurers. This editorial invites neurosurgeons and radiation oncologists to help determine whether enthusiasm has outpaced evidence or whether these efforts, ours and those preceding us back to Leksell, contain substantive promise.

The field now advances from empirical lesioning toward mechanistically informed, image-guided, and data-driven precision. These developments also signal a potential transfer of scientific agency from traditional centers toward a more globally distributed community of innovators.

If Leksell could see the future

If we could return to Stockholm in 1951 and report that, in 2025, neurosurgeons in El Salvador published the world’s first clinical application of connectomic-guided radiosurgery for the treatment of pain [11], mapping and modulating the thalamus through noninvasive precision beams, Leksell might first express disbelief, then satisfaction. He would likely recognize shared principles: minimal invasion, anatomical respect, and precision over force. He might also observe that his instrument, once a Swedish prototype, has become an equalizer, enabling nations with limited surgical infrastructure to perform complex functional interventions safely and respectfully.

Artificial intelligence and the new democratization of science

Artificial intelligence (AI) now amplifies this democratization. Machine learning-assisted planning can predict dose distributions, automate shot selection, and integrate functional connectivity data to individualize treatment [13,14], extending Leksell’s geometry-based precision into an era of data-driven decision-making. At the same time, AI may help reduce the practical barriers that limit radiosurgery access in low- and middle-income countries, where challenges are often structural rather than scientific. In El Salvador, these obstacles included pricing and limited negotiating leverage before consistent outcomes could be demonstrated; a long and often solitary learning curve without ongoing expert companionship; and limited awareness among clinicians and patients regarding the safety and benefits of radiosurgery. Cloud collaboration, automated plan quality benchmarking, and open-access dissemination reduce economic and geographic barriers, fostering a global network of peers rather than a hierarchy of privilege. In parallel, AI-supported workflows, such as automated contouring, dose optimization, and remote case review, can reduce dependence on large specialist teams and provide real-time guidance during treatment planning. In this way, AI may lower the cost and expertise thresholds required to safely deliver functional radiosurgery, enabling centers like those in El Salvador to not only adopt the technique but also contribute meaningfully to its global development.

Opportunities and responsibilities

The resurgence of radiosurgical thalamotomy, and the possibility of radiomodulation by avoiding ablative doses to medial thalamic structures, invites both optimism and skepticism. Outcome variability underscores the need for standardized dosimetry, careful target definition, and long-term follow-up. Radiation lesioning for pain carries risks, including delayed necrosis and sensory deficits [5,6,9], and although generally considered safe, its efficacy and universal acceptance require rigorous establishment.

Adoption of AI similarly demands transparency, validation, and ethical oversight. Algorithms should support, not supplant, clinical judgment, and datasets must include LMIC representation to avoid digital inequity, as is often the case and exemplified by the extreme underrepresentation of less than 3% of neurosurgical publications [15].

Conclusion

Lars Leksell’s thalamotomy for pain began as a scientific rebellion. Seventy-four years later, work in San Salvador affirms that spirit, not by merely reproducing his technique but by extending his philosophy. Functional radiosurgery has evolved from experimental lesioning to a versatile therapeutic platform. Its newest frontier is ethical: making precision neurosurgery truly universal. Were Leksell to see neurosurgeons in El Salvador using the Gamma Knife, AI, and connectomics (the mapping of brain networks) to relieve pain without incision, he might judge his prophecy fulfilled, and still unfolding.
